# Various Coating Methodologies of WO_3_ According to the Purpose for Electrochromic Devices

**DOI:** 10.3390/nano10050821

**Published:** 2020-04-25

**Authors:** Keon-Woo Kim, Yong Min Kim, Xinlin Li, Taehwa Ha, Se Hyun Kim, Hong Chul Moon, Seung Woo Lee

**Affiliations:** 1 School of Chemical Engineering, Yeungnam University, 280 Daehak-ro, Gyeongsan 38541, Korea; 2 Department of Chemical Engineering, University of Seoul, Seoul 02504, Korea; 3 College of Electromechanical Engineering, Qingdao University, Qingdao 266071, China

**Keywords:** electrochromic device, tungsten trioxide, printed electronics, slot-die, electrohydrodynamic jet printing

## Abstract

Solution-processable electrochromic (EC) materials have been investigated widely for various applications, such as smart windows, reflective displays, and sensors. Among them, tungsten trioxide (WO_3_) is an attractive material because it can form a film via a solution process and relative low temperature treatment, which is suitable for a range of substrates. This paper introduces the slot-die and electrostatic force-assisted dispensing (EFAD) printing for solution-processable methods of WO_3_ film fabrication. The resulting films were compared with WO_3_ films prepared by spin coating. Both films exhibited a similar morphology and crystalline structure. Furthermore, three different processed WO_3_ film-based electrochromic devices (ECDs) were prepared and exhibited similar device behaviors. In addition, large area (100 cm^2^) and patterned ECDs were fabricated using slot-die and EFAD printing. Consequently, slot-die and EFAD printing can be used to commercialize WO_3_ based-ECDs applications, such as smart windows and reflective displays.

## 1. Introduction

Solution-processable electronic materials have attracted considerable attention in a range of optoelectronic fields, such as displays [1,2,3], thin-film transistors [4,5,6], and sensors [7,8,9]. This is because their processing advantages (e.g., roll-to-roll [10,11,12] and several printing processes [13,14,15,16]) make it possible to commercialize low-cost and large-area optoelectronic devices. Among them, electrochromic (EC) materials (e.g., metal oxides [3,9,13], metal complexes [17,18], viologens [19,20,21,22,23,24,25], small organic molecules [26,27], metallo-supramolecular polymers [28,29,30,31], and conducting polymer thin films [32,33,34,35]) exhibit a reversible change in optical transmittance in response to an applied external voltage. Therefore, they have been investigated extensively for use in a variety of applications, such as reflective displays, antiglare mirrors, smart windows, and functional supercapacitors.

Tungsten trioxide (WO_3_) is a widely used EC material owing to its facile fabrication method via solution processing, such as a sol-gel technique at relatively low temperatures (~60 °C), which enables the use of conventional plastic substrates including polyethylene terephthaltate (PET) [3,13,36]. To apply WO_3_ in information displays or smart mirror/windows, it is necessary to develop manufacturing techniques to suit the characteristics of the application. Although spin-casting is one representative method to form WO_3_ films and investigate various EC properties in detail [36], there are demands to make shapes or letters for information transfer (display application). In addition, the previous methods may not be efficient for large area manufacturing with respect to film uniformity and quantity of materials consumption during film formation (smart window/mirror application). Therefore, an appropriate methodology is needed to commercialize WO_3_-based EC device (ECD) applications.

This paper introduces slot-die coating and electrohydrodynamic (EHD) jet printing of a WO_3_ ink for a uniform film coating on a large area and patterning, respectively. A slot-die coating method allows the pre-metered and continuous coating of ink flowing from the downstream meniscus, forming at a horizontal slot-die head edge while the ink is supplied consistently in the slot-die head [15,37]. Therefore, it is considered to be a cost-effective and easily scalable technique for the high throughput and large area film production field [38,39,40]. On the other hand, EHD jet printing enables uniform and continuous line patterning by forming a jet stream from the nozzle tip, where the electrostatic force applied between a nozzle tip and a substrate can deform the meniscus of an ink to eject droplets consistently [11,13,14]. Among EHD printing modes, electrostatic force-assisted dispensing (EFAD) mode, which applies very low external voltage between the nozzle tip and substrate for the formation of continuous ink flow between the two, have been frequently applied to make patterns for electronic materials with good pattern fidelity and morphological uniformity [36].

In this study, the coating and morphological properties of WO_3_ films fabricated by slot-die coating and EFAD printing were characterized and compared with films produced by spin-coating. In addition, the EC performance of the device fabricated by each printing method was compared. The ECDs showed similar morphological properties of WO_3_ and device behaviors, such as optical modulation, switching speed, and coloration efficiency. These results imply that the slot-die and EFAD printing can be commercialized for diverse WO_3_-based ECDs applications. To demonstrate the feasibility, the large scale (100 cm^2^) and patterned WO_3_-based ECDs were produced by slot-die and EFAD printing, respectively. The obtained ECDs can be used as smart windows and reflective display applications.

## 2. Materials and Methods

### 2.1. Materials

Except for ITO glass (sheet resistance: 15 Ω/sq, Asahi Glass Co., Tokyo, Japan), all materials were purchased from Sigma-Aldrich (St. Louis, MO, USA). Tungsten trioxide (WO_3_) nanoparticles were prepared using a method reported elsewhere [3,13,14,32]. To synthesize WO_3_ nanoparticles, tungsten (W) powder (7.0 g) was added to hydrogen peroxide solution (31 wt.% in water, 90 g) and allowed to react at 100 °C. After 5 h, the solvent was evaporated by using rotary evaporator, giving WO_3_ nanoparticles. The characterizations of resulting WO_3_ nanoparticles using photograph, scanning electron microscopy (SEM) and X-ray diffraction (XRD) are shown in Figure S1a–c, respectively. A WO_3_ suspension was fabricated by mixing WO_3_ nanoparticles, DI-water, and isopropyl alcohol (IPA) at a weight ratio of 0.3:0.35:0.35, respectively, followed by sonication for 4 h to obtain a homogeneous dispersion. Before WO_3_ suspension deposition, ITO glasses were cleaned sequentially with acetone (15 min) and IPA (15 min) with sonication, and a UV/ozone treatment was then conducted for 10 min prior use. In addition, propylene carbonate containing LiClO_4_ (0.5 M) and ferrocene (0.05 M) was used as an electrolyte.

### 2.2. Fabrication of Tungsten Oxide Film

In this study, three different processes (spin coating, slot-die, and EFAD printing) were used to fabricate WO_3_ films, and the thickness of the WO_3_ films was maintained for a fair comparison. To fabricate the same film thickness, in the case of spin coating, the WO_3_ suspension was spin-coated on ITO at 5000 rpm for 20 s. For the slot-die process, a WO_3_ suspension was ejected into the slot-die head at a flow rate of 1 mL/min, and the moving speed was set to 5 mm/s. In EFAD printing, the flow rate of the WO_3_ suspension was fixed to 0.5 µL/min, and the applied voltage between the nozzle tip and substrate and printing velocity were 10 V and 3 mm/s, respectively. To prepare WO_3_ films with a size of 1.5 cm × 1.5 cm using spin-coating, slot-die and EFAD printing, the WO_3_ suspension required approximately 100 µL, 1 µL and 1 µL, respectively. Prior to use, the as-spun WO_3_ films were annealed thermally at 60 °C under vacuum for 10 h.

### 2.3. Device Fabrication and Characterization

The WO_3_ films obtained by the three different processes were characterized by optical microscopy (OM, ECLIPSE LV100ND, Nikon, Tokyo, Japan), scanning electron microscopy (SEM, S-4800, Hitachi, Tokyo, Japan), X-ray diffraction (XRD, D/MAX2500 VL-PC, Rigaku, Tokyo, Japan), and cyclic voltammetry (Weis 500, WonA Tech., Seoul, Korea). For WO_3_-based ECDs assembly, the WO_3_-coated ITO glass, electrolyte solution, and counter bare ITO glass were sandwiched to form the (ITO/WO_3_/electrolyte/ITO) configuration, in which ~88 µm thick double-sided tape was used as a spacer and adhesive. To investigate the performance of ECDs, DC and square-shaped wave voltages were supplied by a potentiostat (Wave Driver 10, Pine Instrument, Durham, NC, USA). In addition, a UV–VIS spectrophotometer (V-730, Jasco, Easton, MD, USA) was used to record the change in transmittance according to the applied voltages. All ECDs in this work were fabricated to a size of 1.5 cm × 1.5 cm to measure characteristics.

## 3. Results

**Figure 1:**
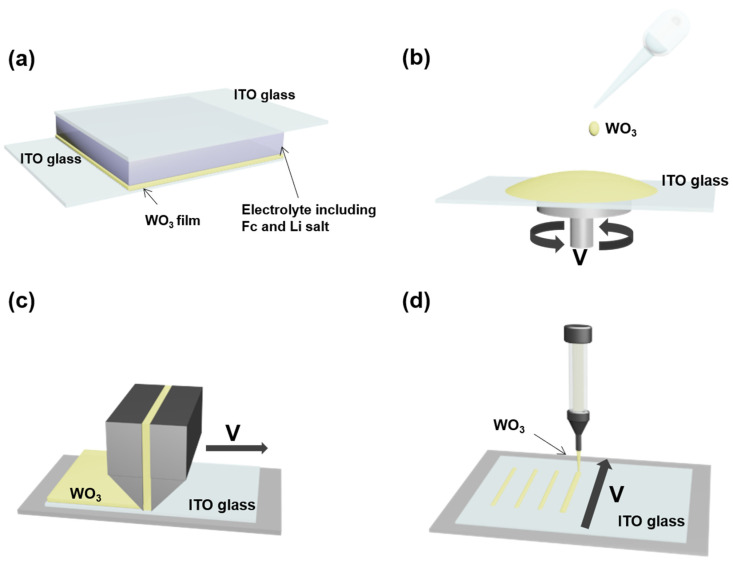
(**a**) Device configuration of the electrochromic device (ECD) in this study. Schematic illustrations of the WO_3_ film fabrication processes in this study (**b**) spin coating, (**c**) slot-die, and (**d**) electrostatic force-assisted dispensing (EFAD) printing.

Figure 1 presents the WO_3_-based ECD configuration and schematic descriptions of various WO_3_ deposition processes (spin coating, slot-die, and EFAD printing) in this study. EC devices based on WO_3_-coated ITO glass were fabricated using propylene carbonate (PC) as an electrolyte containing 0.5 M LiClO_4_ and 0.05 M ferrocene (Fc) (Figure 1a). In this device, the ion storage layer was unnecessary because Fc acted as anodic species. Thus, the device configuration became simple. In the spin-coating process (Figure 1b), the ITO glass should be fixed on a vacuum chamber, followed by casting the WO_3_ suspension on ITO glass and spinning the chamber to form a uniform WO_3_ film. Although spin coating is a facile method to fabricate the films precisely, it is limited to large area and patterning productions because of the necessity of large amounts of solution and additional post-process. The slot-die and EFAD printing are attractive technologies that allow the fabrication of large area and patterned films by the simple direct printing of a solution. Figure 1c,d show schematic illustrations of the slot-die and EFAD printing processes, respectively. The slot-die head was placed vertically at ITO glass, and the WO_3_ suspension was injected through a connected tube. The WO_3_ suspension was ejected from slot-die head to ITO glass, while the slot-die head was moved horizontally to form a uniform large-area WO_3_ film. In EFAD printing, the WO_3_ suspension was filled into a syringe with a nozzle.

An electric field between the nozzle tip and ITO glass was applied to enable well-defined and continuous line printing while moving the sample stage. The three different films (obtained by spin coating, slot-die, and EFAD printing) underwent thermal annealing at 60 °C under vacuum prior to use.

**Figure 2:**
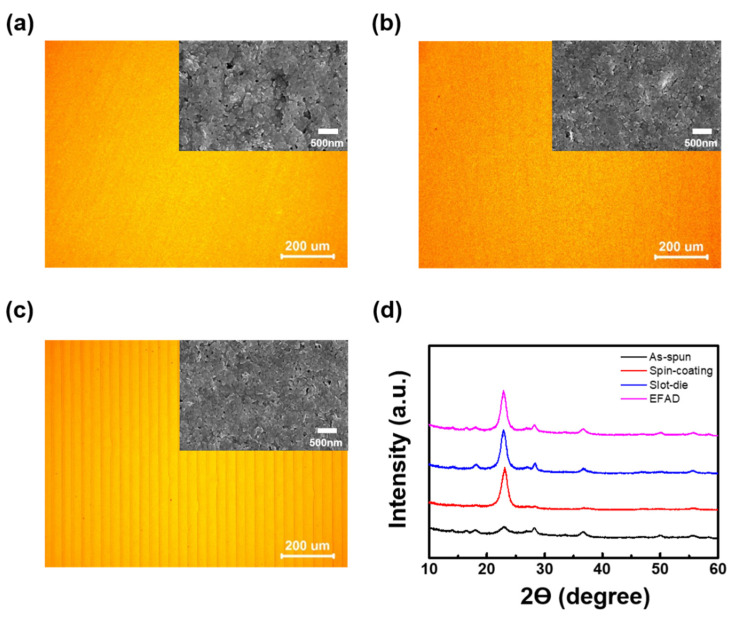
Optical microscopy (OM) and scanning electron microscopy (SEM) (see in inset) images of WO_3_ films morphology obtained by (**a**) spin-coating, (**b**) slot-die and (**c**) EFAD printing. (**d**) X-ray diffraction (XRD) patterns of the WO_3_ films, as-spun, and after thermal annealing at 60 °C under vacuum.

The WO_3_ film morphologies obtained by three different processes were investigated by OM, as shown in Figure 2a–c (see the SEM images in the inset). The thickness of three WO_3_ films were determined to be ~300 nm (see cross-section SEM images in Figure S2). OM showed that all the films except for the EFAD film exhibited a similar shape. The EFAD film showed an overlapped line pattern because it needed to be printed several times to produce the same film thickness as the spin-coating and slot-die films. Although the OM image of the EFAD film showed an overlapping line pattern, SEM revealed the three different films to have a similar morphology. Specifically, electrochromic performance is governed by the crystalline structure of WO_3_. Therefore, the crystalline structure of the WO_3_ films (spin coating, slot-die, and EFAD) was examined by XRD. Figure 2d shows the XRD patterns of the WO_3_ films before and after thermal annealing. The as-spun WO_3_ film (before thermal annealing) showed XRD reflections corresponding to the (002), (200), and (202) planes. After thermal annealing, each WO_3_ film (spin coating, slot-die, and EFAD) exhibited an enhanced (002) peak intensity, indicating the development of the monoclinic crystalline structure of WO_3_. Therefore, the electrochromic (EC) performance of each WO_3_ film-based ECD was expected to be similar because of their comparable film morphology and crystalline structure.

**Figure 3:**
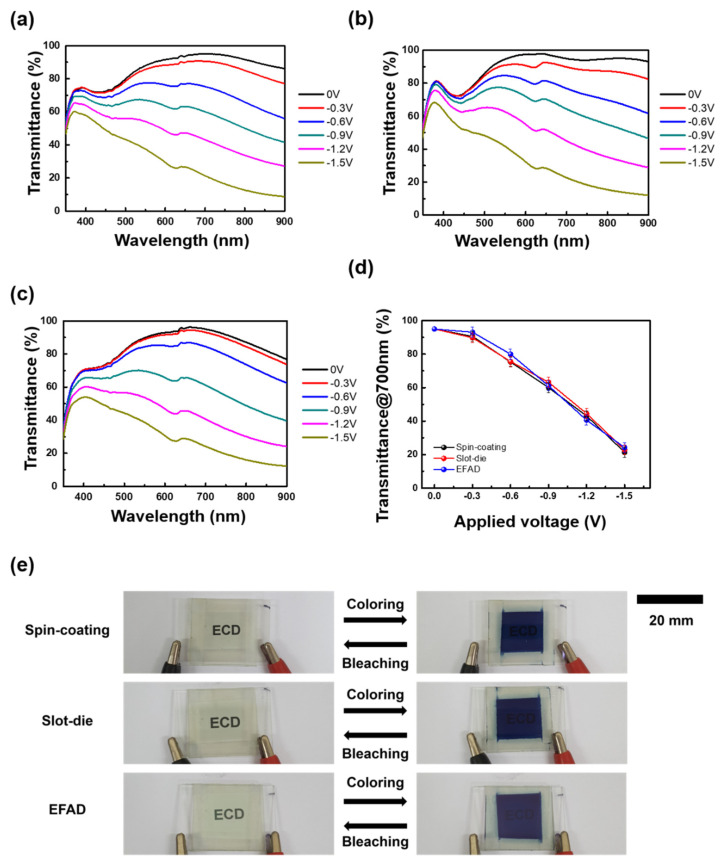
Variation in the UV–VIS spectra for ECDs obtained by three different processes (**a**) spin-coating, (**b**) slot-die, and (**c**) EFAD printing. (**d**) Voltage dependence of the transmittance at 700 nm. (**e**) Photograph of the original state (no bias), colored state (−1.5 V), and bleached state (0 V). The scale bar represents 20 mm.

The cyclic voltammograms (CVs) of WO_3_-coated films fabricated by three different methods were recorded at a scan rate of 25 mV/s from +1.0 to −1.5 V to estimate the electrochemical properties and electrochromic performances of the films (Figure S3). When applied to a negative potential, significant increasing current densities were measured at each film indicating an activation process for the intercalation of Li^+^ ions into the films. Despite the different coating methods, the shapes of the CV curves were similar for all the coating methods. To examine the EC behaviors of the three different WO_3_ film-based ECDs, the transmittance variations, according to the applied voltage, were recorded at 350–900 nm (Figure 3a–c). A noticeable decrease in the transmittance spectra was observed at −0.3 V, and the transmittance spectra of ECDs according to applied voltage exhibited similar behavior. The color changes at increasing bias were also analyzed with CIELAB color coordinates. In the bleached state the films had a slight yellowish color with L^*^, a^*^ and b^*^ of each device are (72.26, 7.67, 16.8), (76.72, 6.24, 8.95), and (71.33, 7.68, 15.21) for spin-coated, slot-die coated, and EFAD films (Figure S4a–c). When increasing the applied voltage until −1.5 V, the devices become blue with the similar values of L^*^, a^*^ and b^*^. In addition, the transmittance variations at 700 nm of the three different WO_3_ film-based ECDs as a function of the applied voltage were derived (Figure 3d). As the applied voltage was increased, the transmittance decreased, and Δ*T* of the three different WO_3_ film-based ECDs were similar 71.3%, 72.8%, and 72.1% at −1.5 V, respectively. The optical transitions of each ECD were also observed clearly, as shown in Figure 3e.

**Figure 4:**
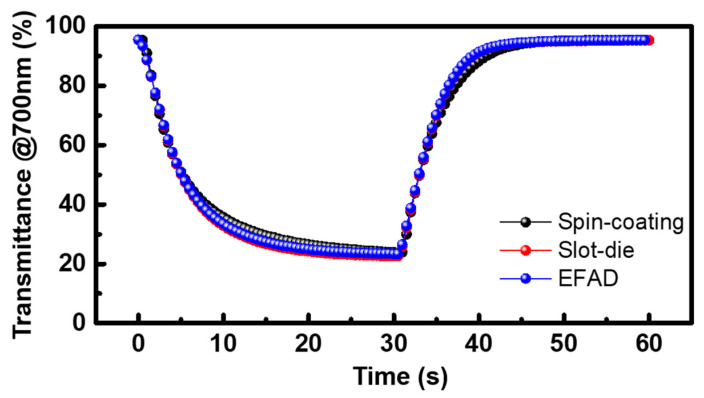
Transmittance profile at 700 nm for ECDs fabricated by three different processes upon the application of −1.5 V (coloration) and 0 V (bleaching).

**Figure 5:**
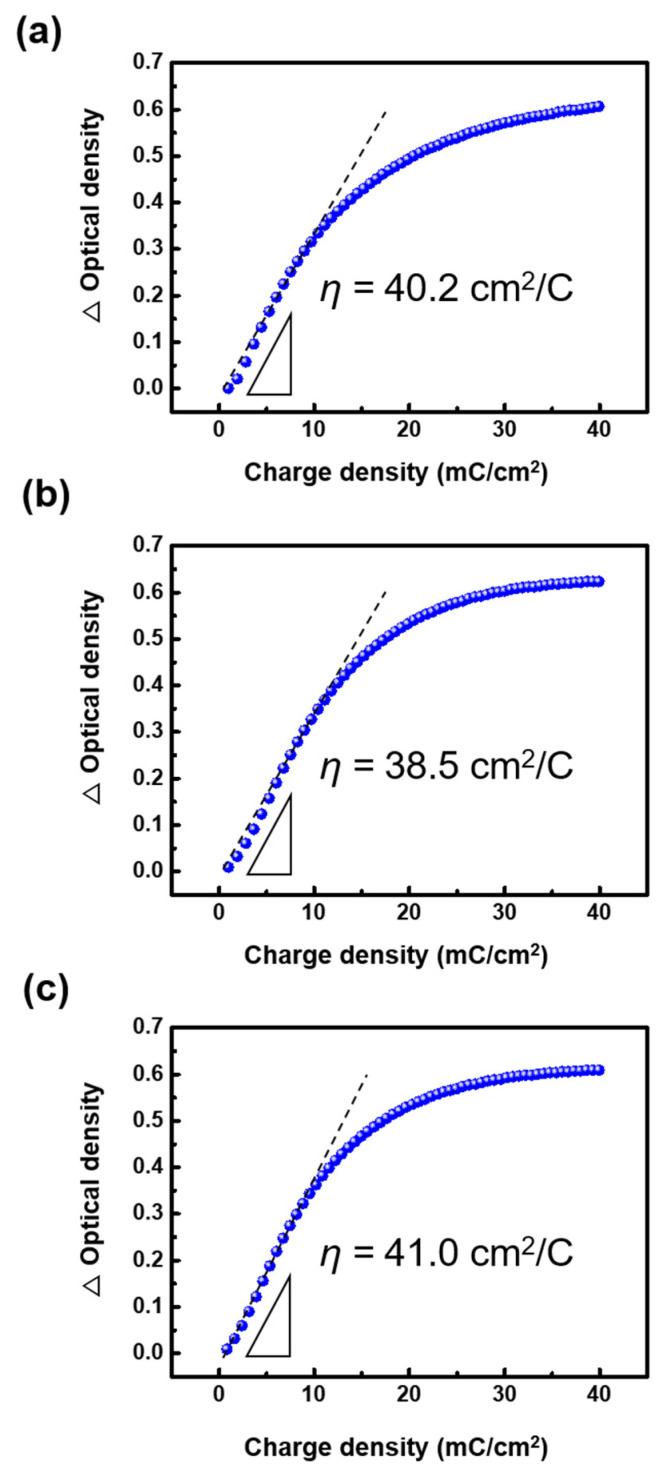
Plots of the optical density as a function of the charge density for three different processes ECDs (**a**) spin-coating, (**b**) slot-die, and (**c**) EFAD printing.

The EFAD film-based ECD showed slightly different dynamic behavior compared to the spin coating and slot-die film based ECDs. To measure the dynamic device behavior, the transmittance profiles of the ECDs were recorded at 700 nm upon the application of −1.5 V (coloration) and 0 V (bleaching) (Figure 4). The response times of coloration (*t_c_*) and bleaching (*t_b_*) were defined as the times at which 90% of the maximum transmittance contrast (Δ*T*) was achieved. The similar coloration (*t_c_*) and bleaching (*t_b_*) times were obtained as *t_c_* = ~14 s and *t_b_* = ~10 s (spin-coating), *t_c_* = ~12 s and *t_b_* = 8.5 s (slot-die) and *t_c_* = ~12 s and *t_b_* = 9 s (EFAD printing). To examine the coloration efficiency (*η*) of the three different ECDs, the correlation between the optical density (*OD*) and charge density (*Q*) was plotted, as shown in Figure 5. The *η* value, which is defined as Δ*OD*/Δ*Q*, corresponds to the slope of the linear fit in the linear regime. The similar *η* values of each ECD were recorded as ~40.2 (spin-coating), ~38.5 (slot-die), and ~41.0 cm^2^/C (EFAD printing).

**Figure 6:**
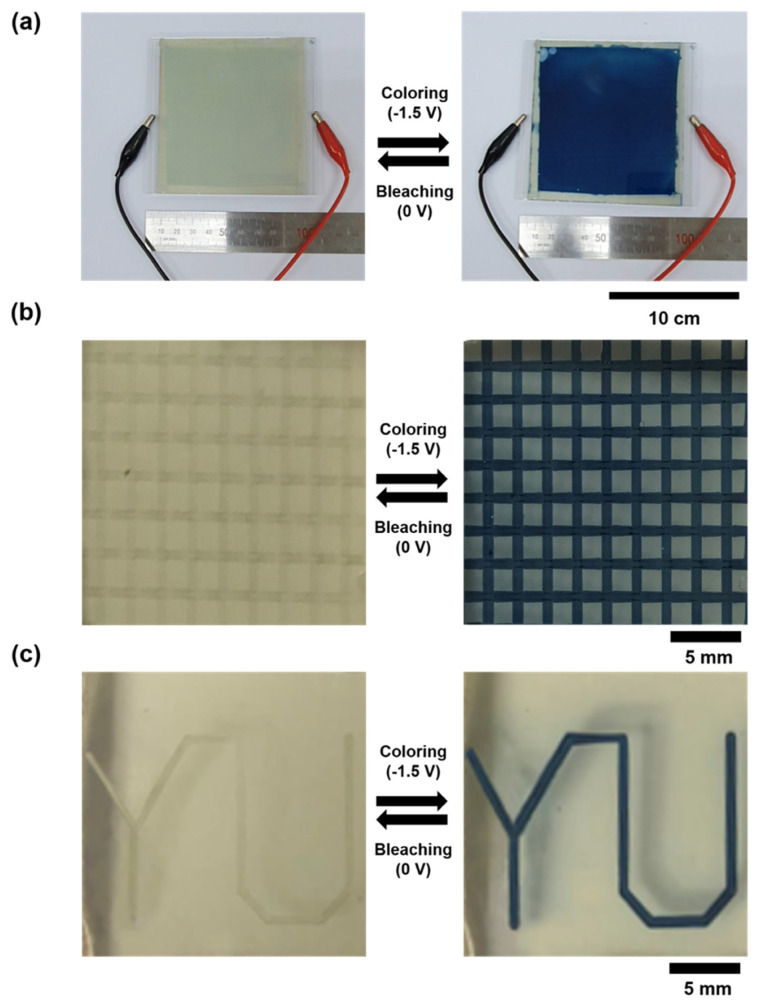
Photographs of the bleached and colored states of WO_3_-based ECDs fabricated by (**a**) slot-die and (**b**,**c**) EFAD printing.

By applying the advantages of slot-die and EFAD printing, which can print a WO_3_ suspension on a large and selective area, large area (100 cm^2^) and patterned WO_3_-based ECDs were obtained, as shown in Figure 6. The color of the large area WO_3_-based ECD changed reversibly over the entire area when the bleached state and colored state were observed at 0 V and −1.5 V, respectively (Figure 6a). Reversible EC behavior was observed in the line patterned WO_3_-based ECD (Figure 6b). In addition, electrochromic letters (YU) were produced using the EFAD printing method (Figure 6c). Accordingly, slot-die and EFAD printing can be used to commercialize WO_3_-based ECD applications, such as smart windows and reflective displays.

## 4. Conclusions

WO_3_-based ECDs were fabricated by spin coating, slot-die, and EFAD printing techniques. The morphology of the WO_3_ films obtained by the above three processes showed a similar shape, except for the EFAD film, which showed an overlapping line. Although the EFAD film exhibited a different morphology, the crystalline structure was no different compared to spin coating and slot-die, which has a monoclinic structure. In addition, the device performance of three different WO_3_-based ECDs was similar. For example, each WO_3_-based ECD produced from spin-coating, slot-die, and EFAD printing showed similar optical modulation (~71.3%, ~72.8%, and ~72.1%), response times (*t_c_* = ~14, ~12, ~12 s), and coloration efficiencies (~40.2, ~38.5, ~41.0 cm^2^/C). Large area (100 cm^2^) and patterned WO_3_ electrochromic devices were demonstrated by taking advantage of the slot-die and EFAD printing processing methods. Slot-die and EFAD printing are attractive technologies for commercializing WO_3_-based ECDs into smart windows and reflective displays.

## Supplementary Materials

The following are available online at https://www.mdpi.com/2079-4991/10/5/821/s1, Figure S1: Information of the WO_3_ nanoparticles; Figure S2: Cross-section SEM images of WO_3_ films; Figure S3: Cyclic voltammograms (CVs) of WO_3_ films; Figure S4: Variations in CIELAB color coordinates of the WO_3_-based ECDs.

## References

[1] Ricciardulli A.G., Blom P.W.M. (2020). Solution-Processable 2D Materials Applied in Light-Emitting Diodes and Solar Cells. Adv. Mater. Technol..

[2] Duan L., Hou L., Lee T.-W., Qiao J., Zhang D., Dong G., Wang L., Qiu Y. (2010). Solution Processable Small Molecules for Organic Light-Emitting Diodes. J. Mater. Chem..

[3] Zhong C., Duan C., Huang F., Wu H., Cao Y. (2011). Materials and Devices toward Fully Solution Processable Organic Light-Emitting Diodes. Chem. Mater..

[4] Gong Y., Zhao K., He H., Cai W., Tang N., Ning H., Wu S., Gao J., Zhou G., Lu X. (2018). Solution Processable High Quality ZrO_2_ Dielectric films for Low Operation Voltage and Flexible Organic Thin Film Transistor Applications. J. Phy. D Appl. Phys..

[5] Rullyani C., Sung C.-F., Lin H.-C., Chu C.-W. (2018). Flexible Organic Thin Film Transistors Incorporating a Biodegradable CO_2_-Based Polymer as the Substrate and Dielectric Material. Sci. Rep..

[6] Jiang L., Li J., Huang K., Li S., Wang Q., Sun Z., Mei T., Wang J., Zhang L., Wang N. (2017). Low-Temperature and Solution-Processable Zinc Oxide Transistors for Transparent Electronics. ACS Omega.

[7] Kim Y.M., Moon H.C. (2020). Ionoskins: Non-volatile, Highly Transparent, Ultra-Stretchable Ionic Sensory Platforms for Wearable Electronics. Adv. Funct. Mater..

[8] Yan Q., Gao L., Tang J., Liu H. (2019). Flexible and Stretchable Photodetectors and Gas Sensors for Wearable Healthcare based on Solution-Processable Metal Chalcogenides. J. Semicond..

[9] Kim S., Le T.-H., Park C.S., Park G., Kim K.H., Kim S., Kwon O.S., Lim G.T., Yoon H. (2017). A Solution-Processable, Nanostructured, and Conductive Graphene/Polyaniline Hybrid Coating for Metal-Corrosion Protection and Monitoring. Sci. Rep..

[10] Liang H.-L., Bay M.M., Vadrucci R., Barty-King C.H., Peng J., Baumberg J.J., Volder M.F.L.D., Vignolini S. (2018). Roll-to-Roll Fabrication of Touch-Responsive Cellulose Photonic Laminates. Nat. Commun..

[11] Tong S., Yuan J., Zhang C., Wang C., Liu B., Shen J., Xia H., Zou Y., Xie H., Sun J. (2018). Large-scale Roll-to-roll Printed, Flexible and Stable Organic Bulk Heterojunction Photodetector. NPJ Flex. Electron..

[12] Espinosa N., Dam H.F., Tanenbaum D.M., Andreasen J.W., Jørgensen M., Krebs F.C. (2011). Roll-to-Roll Processing of Inverted Polymer Solar Cells using Hydrated Vanadium (V) Oxide as a PEDOT:PSS Replacement. Materials.

[13] Polat E.O., Balcı O., Kocabas C. (2015). Graphene based Flexible Electrochromic Devices. Sci. Rep..

[14] Zhang S., Chen S., Hu F., Xu R., Yan B., Jiang M., Gu Y., Yang F., Cao Y. (2019). Spray-Processable, Large-Area, Patterned and All-Solid-State Electrochromic Device based on Silica/Polyaniline Nanocomposites. Sol. Energy Mater. Sol. Cells.

[15] Bellingham A., Bromhead N., Fontecchio A. (2017). Rapid Prototyping of Slot Die Devices for Roll to Roll Production of EL Fibers. Materials.

[16] Zhang Z., Guo K., Li Y., Li X., Guan G., Li H., Luo Y., Zhao F., Zhang Q., Wei B. (2015). A Colour-Tunable, Weavable Fibre-Shaped Polymer Light-Emitting Electrochemical Cell. Nature.

[17] Chernova N.A., Roppolo M., Dillon A.C., Whittingham M.S. (2009). Layered Vanadium and Molybdenum Oxides: Batteries and Electrochromics. J. Mater. Chem..

[18] Deb S.K. (2008). Opportunities and challenges in science and technology of WO_3_ for electrochromic and related applications. Sol. Energy Mater. Sol. Cells.

[19] Moon H.C., Kim C.-H., Lodge T.P., Frisbie C.D. (2016). Multicolored, Low Power, Flexible Electrochromic Devices Based on Ion Gels. ACS Appl. Mater. Interfaces.

[20] Kao S.-Y., Kawahara Y., Nakatsuji S., Ho K.-C. (2015). Achieving a Large Contrast, Low Driving Voltage, and High Stability Electrochromic Device with a Viologen Chromophore. J. Mater. Chem. C.

[21] Wang X., Guo L., Cao S., Zhao W. (2020). Highly Stable Viologens-Based Electrochromic Devices with Low Operational Voltages Utilizing Polymeric Ionic Liquids. Chem. Phys. Lett..

[22] Li G., Zhang B., Wang J., Zhao H., Ma W., Xu L., Zhang W., Zhou K., Du Y., He G. (2019). Electrochromic Poly(chalcogenoviologen)s as Anode Materials for High-Performance Organic Radical Lithium-Ion Batteries. Angew. Chem..

[23] Kim J.-W., Myoung J.-M. (2019). Flexible and Transparent Electrochromic Displays with Simultaneously Implementable Subpixelated Ion Gel-Based Viologens by Multiple Patterning. Adv. Funct. Mater..

[24] Kao S.-Y., Lu H.-C., Kung C.-W., Chen H.-W., Chang T.-H., Ho K.-C. (2016). Thermally Cured Dual Functional Viologen-Based All-in-One Electrochromic Devices with Panchromic Modulation. ACS Appl. Mater. Interfaces.

[25] Sydam R., Deepa M., Joshi A.G. (2013). A Novel 1,1′-bis[4-(5,6-dimethyl-1*H*-benzimidazole-1-yl)butyl]-4,4′-bipyridinium dibromide (Viologen) for a High Contrast Electrochromic Device. Org. Eletcon..

[26] Li R., Li K., Wang G., Li L., Zhang Q., Yan J., Chen Y., Zhang Q., Hou C., Li Y. (2018). Ion-Transport Design for High-Performance Na^+^-Based Electrochromics. ACS Nano.

[27] Mortimer R.J. (1999). Organic Electrochromic Materials. Electrochim. Acta.

[28] Roy S., Chakraborty C. (2019). Nanostructured Metallo-Supramolecular Polymer-based Gel-type Electrochromic Devices with Ultrafast Switching Time and High Colouration Efficiency. J. Mater. Chem. C.

[29] Han F.S., Higuchi M., Kurth D.G. (2007). Metallo-Supramolecular Polymers Based on Functionalized Bis-terpyridines as Novel Electrochromic Materials. Adv. Mater..

[30] Higuchi M., Kurth D.G. (2007). Electrochemical functions of metallosupramolecular nanomaterials. Chem. Rec..

[31] Han F.S., Higuchi M., Kurth D.G. (2008). Metallosupramolecular Polyelectrolytes Self-Assembled from Various Pyridine Ring-Substituted Bisterpyridines and Metal Ions: Photophysical, Electrochemical, and Electrochromic Properties. J. Am. Chem. Soc..

[32] Honda K., Fujita M., Ishida H., Yamamoto R., Ohgaki K. (1988). Solid-State Electrochromic Devices Composed of Prussian Blue, WO_3_, and Poly (ethylene oxide)-Polysiloxane Hybrid-Type Ionic Conducting Membrane. J. Electrochem. Soc..

[33] Jiang M., Zhao Z.F. (1990). Novel Stable Electrochromic Thin Film: A Prussian Blue Analogue based on Palladium Hexacyanoferrate. J. Electroanal. Chem..

[34] Shirota Y. (2000). Organic materials for electronic and optoelectronic devices. J. Mater. Chem..

[35] Heuer H.W., Wehrmann R., Kirchmeyer S. (2002). Electrochromic Window Based on Conducting Poly(3,4-ethylenedioxythiophene)–Poly(styrene sulfonate). Adv. Funct. Mater..

[36] Li X., Yun T.Y., Kim K.-W., Kim S.H., Moon H.C. (2020). Voltage-Tunable Dual Image of Electrostatic Force-Assisted Dispensing Printed, Tungsten Trioxide-Based Electrochromic Devices with a Symmetric Configuration. ACS Appl. Mater. Interfaces.

[37] Jensen J., Dam H.F., Reynolds J.R., Dyer A.L., Krebs F.C. (2012). Manufacture and Demonstration of Organic Photovoltaic-Powered Electrochromic Displays using Roll Coating Methods and Printable Electrolytes. J. Polym. Sci. B Polym. Phys..

[38] Liu H.-S., Chang W.-C., Chou C.-Y., Pan B.-C., Chou Y.-S., Liou G.-S., Liu C.-L. (2017). Controllable Electrochromic Polyamide Film and Device Produced by Facile Ultrasonic Spray-coating. Sci. Rep..

[39] Cai G.F., Cui M.Q., Kumar V., Darmawan P., Wang J.X., Wang X., Eh A.L.-S., Qian K., Lee P.S. (2016). Ultra-large Optical Modulation of Electrochromic Porous WO_3_ film and the Local Monitoring of Redox Activity. Chem. Sci..

[40] Layani M., Darmawan P., Foo W.L., Liu L., Kamyshny A., Mandler D., Magdassi S., Lee P.S. (2014). Nanostructured Electrochromic films by Inkjet Printing on Large Area and Flexible Transparent Silver Electrodes. Nanoscale.

