# Supplementary material for: Various Coating Methodologies of WO3 According to the Purpose for Electrochromic Devices
Source: Nanomaterials (Basel). 2020 Apr 25;10(5):821. doi: 10.3390/nano10050821 (PMC7711473; doi:10.3390/nano10050821)
Supplement: Supplementary file 1 [file nanomaterials-10-00821-s001.pdf]

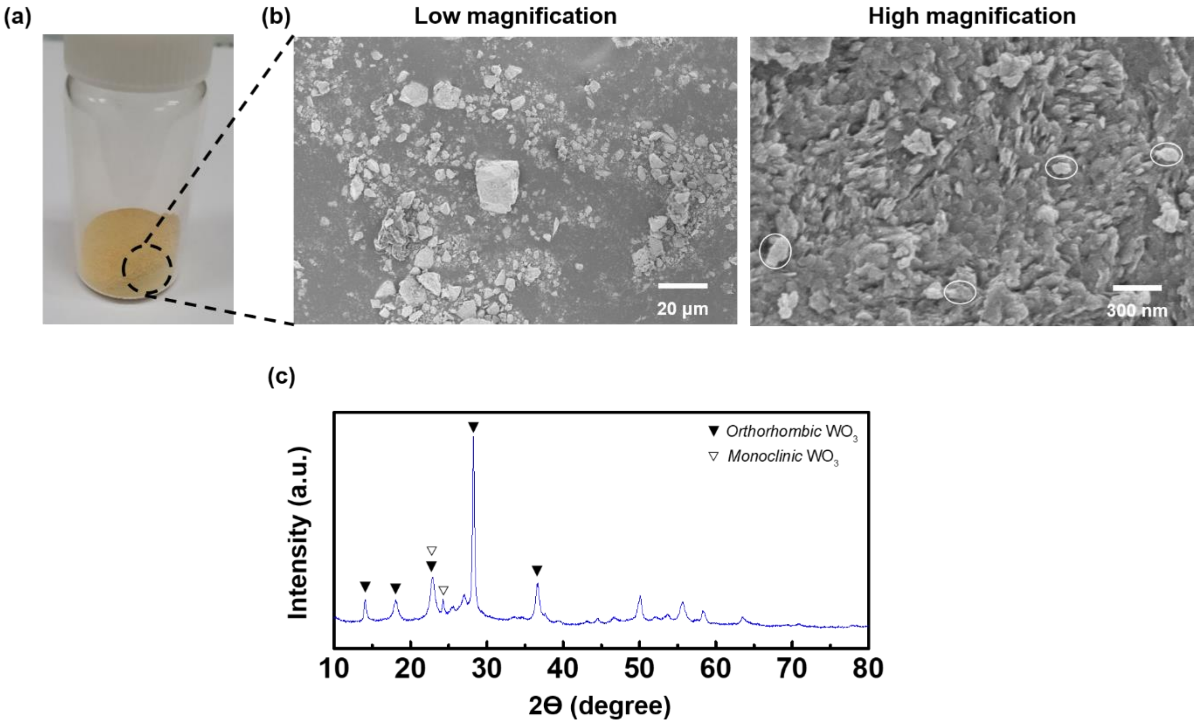

Figure S1. (**a**) Photograph of WO_3 nanoparticles. The WO_3 nanoparticles exhibits yellow color corresponded to its oxidation state (W^{+6}). (**b**) SEM images of the WO_3 nanoparticles. The initial state of the WO_3 nanoparticles are agglomerated and have irregular oval shape with a size of about ~100 nm. (**c**) The XRD patterns of WO_3 nanoparticles, showing that the mixed state of orthorhombic and monoclinic crystalline structure.

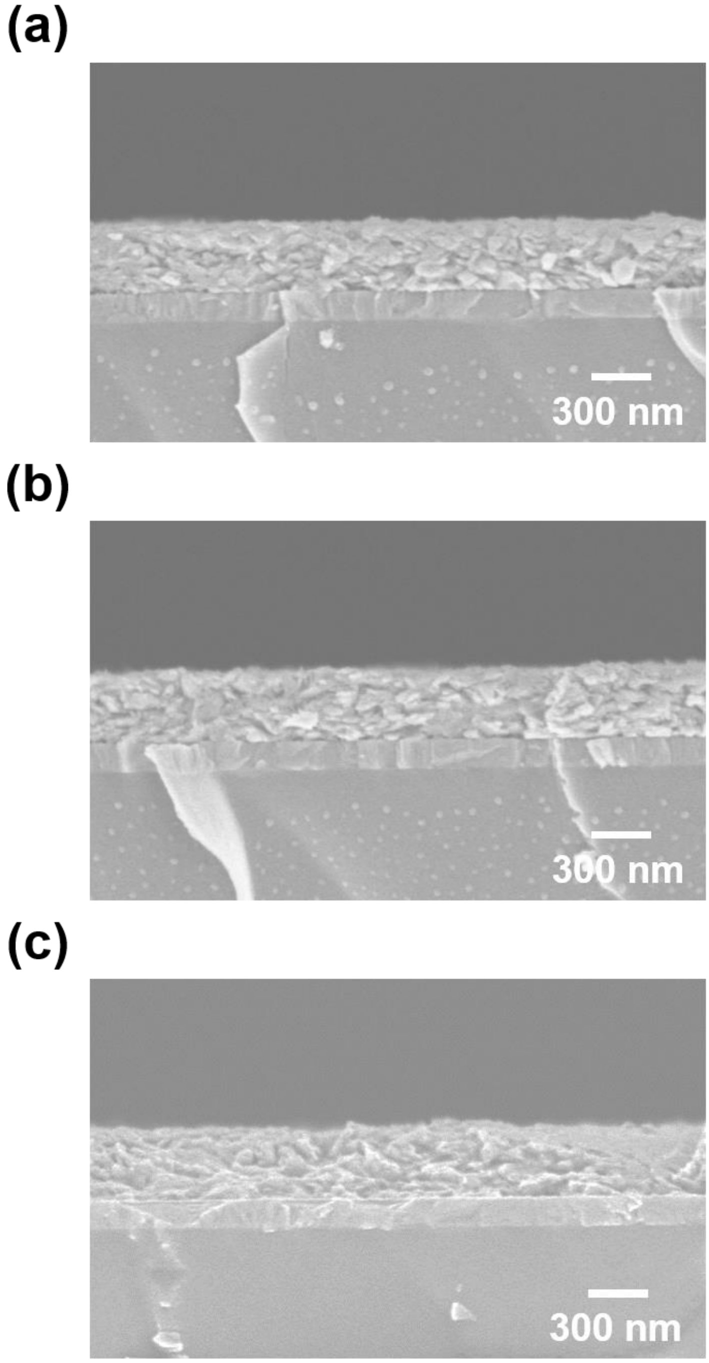

Figure S2. Cross-section SEM images of WO_3 films obtained by (**a**) spin-coating, (**b**) slot-die and (**c**) EFAD printing.

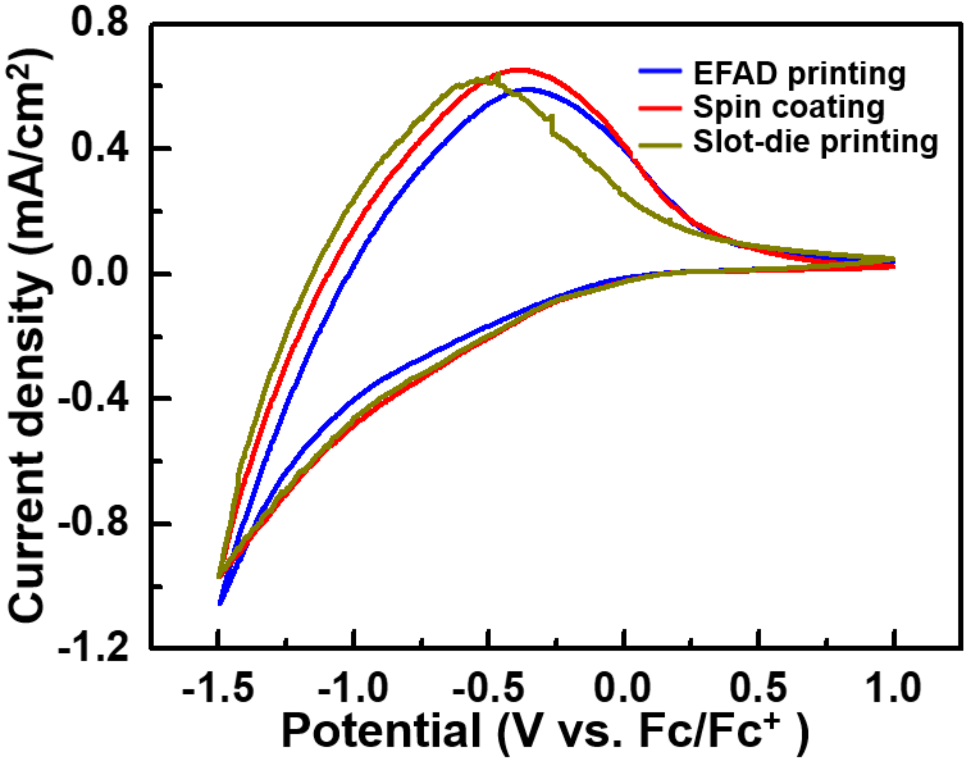

Figure S3. Cyclic voltammograms (CVs) of WO_3 films fabricated by three different methods were measured at a scan rate of 25 mV/s, where platinum disk, Ag wire were employed as the counter, reference electrode, respectively. Propylene carbonate (PC) solution containing 0.5 M $LiClO_4$ was used as electrolyte.

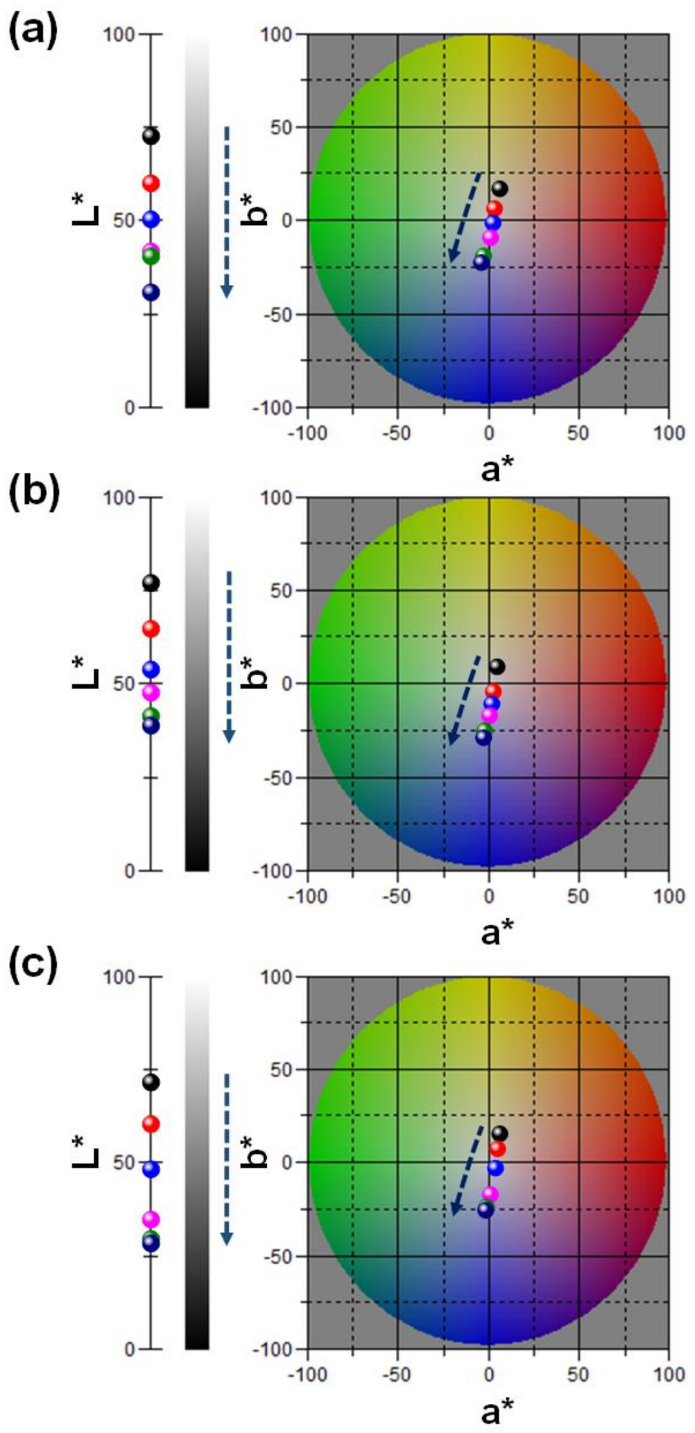

Figure S4. Variations in CIELAB color coordinates (L^*, $a^*$and b^*) of the WO_3 based ECDs which prepared by (**a**) spin-coating, (**b**) slot-die and (**c**) EFAD printing, respectively.
